# Supplementary material for: Themis2 regulates natural killer cell memory function and formation
Source: Nat Commun. 2023 Nov 8;14:7200. doi: 10.1038/s41467-023-42578-8 (PMC10632368; doi:10.1038/s41467-023-42578-8)
Supplement: Supplementary file 1 — Supplementary Information [file 41467_2023_42578_MOESM1_ESM.pdf]

Supplementary Figures

Themis2 regulates natural killer cell memory function and formation

Tsukasa Nabekura[1,2,3]*, Elfira Amalia Deborah[2,4], Saeko Tahara[2,5,6], Yuya Arai[2,6,7], Paul E. Love[8], Koichiro Kako[1,9], Akiyoshi Fukamizu[1], Masafumi Muratani[10], and Akira Shibuya[1,2,3]*

[1]Life Science Center for Survival Dynamics, Tsukuba Advanced Research Alliance (TARA), University of Tsukuba, Ibaraki, 305-8575, Japan.

[2]Department of Immunology, Faculty of Medicine, University of Tsukuba, Ibaraki, 305-8575, Japan.

[3]R&D Center for Innovative Drug Discovery, University of Tsukuba, Ibaraki, 305-8575, Japan.

[4]Doctoral Program in Medical Sciences, Graduate School of Comprehensive Human Sciences, University of Tsukuba, Ibaraki, 305-8575, Japan.

[5]College of Medicine, School of Medicine and Health Sciences, University of Tsukuba, Ibaraki, 305-8575, Japan

[6]Bioinformatics Laboratory, Faculty of Medicine, University of Tsukuba, Ibaraki, 305-8575, Japan

[7]College of Biological Sciences, School of Life and Environmental Sciences, University of Tsukuba, Ibaraki, 305-8575, Japan

[8]Section on Hematopoiesis and Lymphocyte Biology, Eunice Kennedy Shriver National Institute of Child Health and Human Development, National Institutes of Health, Bethesda, MD 20892, U.S.A.

[9]Faculty of Life and Environmental Sciences, University of Tsukuba, Ibaraki, 305-8575, Japan.

[10]Department of Genome Biology, Faculty of Medicine, University of Tsukuba, Ibaraki, 305-8575, Japan.

*Corresponding authors:

Akira Shibuya, MD, PhD, ashibuya@md.tsukuba.ac.jp

Tsukasa Nabekura, PhD, nabekura.tsukasa.fe@u.tsukuba.ac.jp

Supplementary Fig. 1

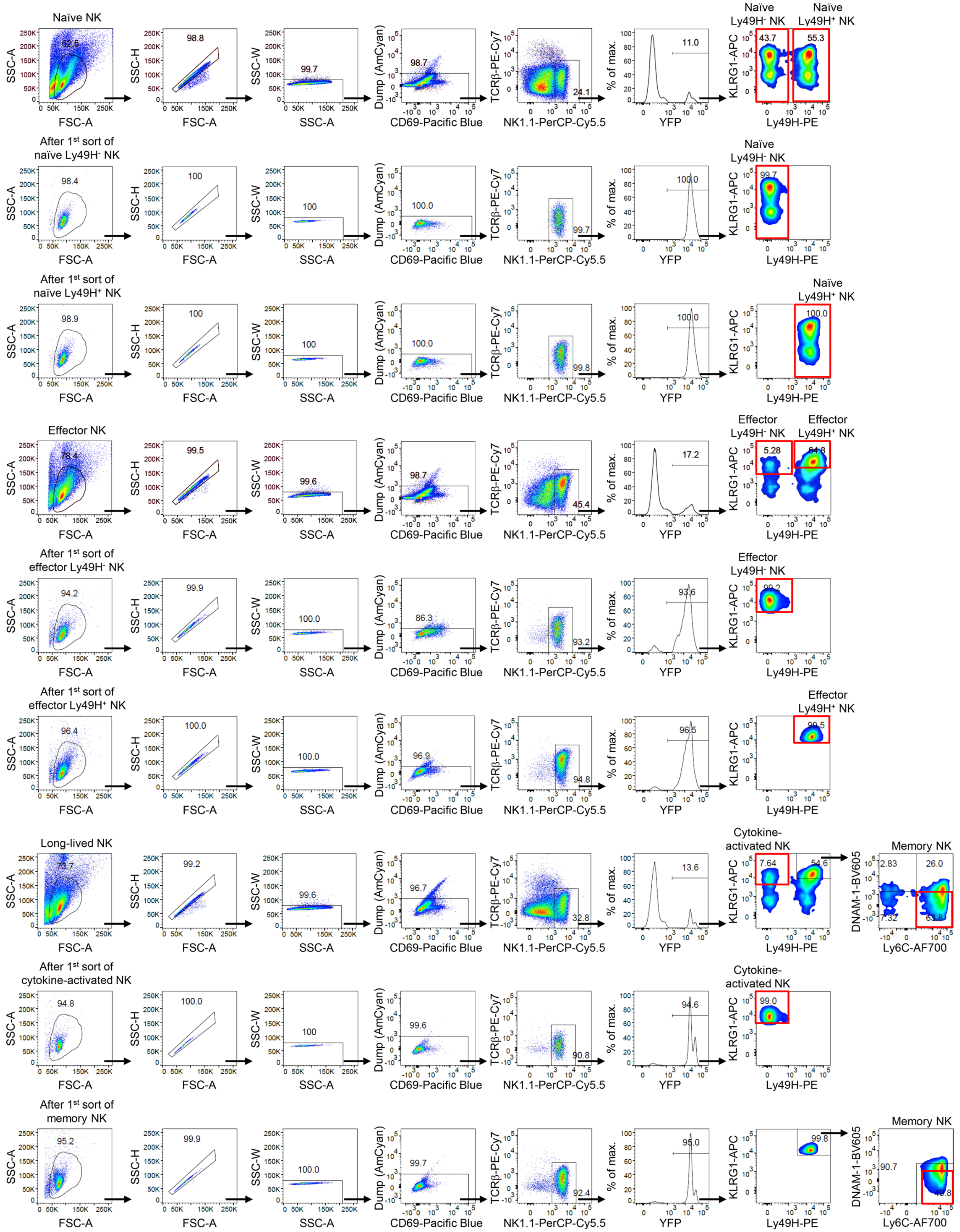

Supplementary Fig. 1. Gating strategy for NK cell subsets.

Gating strategies for naïve, effector, and long-lived natural killer (NK) cell subsets expressing or not expressing Ly49H. Naïve Ly49H^{+} and Ly49H^{-} NK cells (yellow fluorescent protein (YFP)$^{+}$NK1.1^{+}TCRβ^{-}Ly49H^{+} or Ly49H^{-} lymphocytes) were purified from the spleen of naïve NK-CreERT2 mice. Effector Ly49H^{+} NK cells (YFP^{+}NK1.1^{+}TCRβ^{-}Ly49H^{+}KLRG1^{high} lymphocytes), effector Ly49H^{-} NK cells (YFP^{+}NK1.1^{+}TCRβ^{-}Ly49H^{-}KLRG1$^{+\text{ to high}}$ lymphocytes), long-lived memory Ly49H^{+} NK cells (YFP^{+}NK1.1^{+}TCRβ^{-}Ly49H^{+}KLRG1^{high}Ly6C^{-}DNAM-1$^{-\text{ to low}}$ lymphocytes), and long-lived cytokine-activated Ly49H^{-} NK cells (YFP^{+}NK1.1^{+}TCRβ^{-}Ly49H^{-}KLRG1$^{+\text{ to high}}$ lymphocytes) were purified from spleen from mouse cytomegalovirus (MCMV)-infected NK-CreERT2 mice. Red boxes represent the gating for the second sorting of individual NK cell subsets. Flow cytometry plots are representative of 2 experiments (*n* = 3 mice in each group).

Supplementary Fig. 2

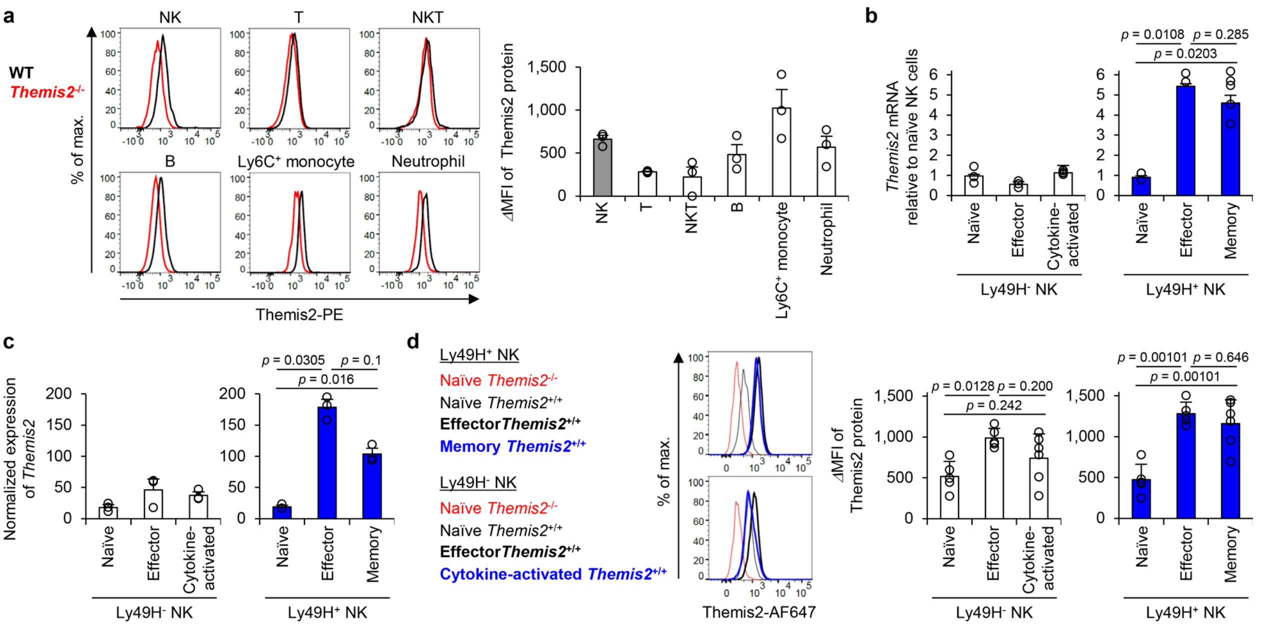

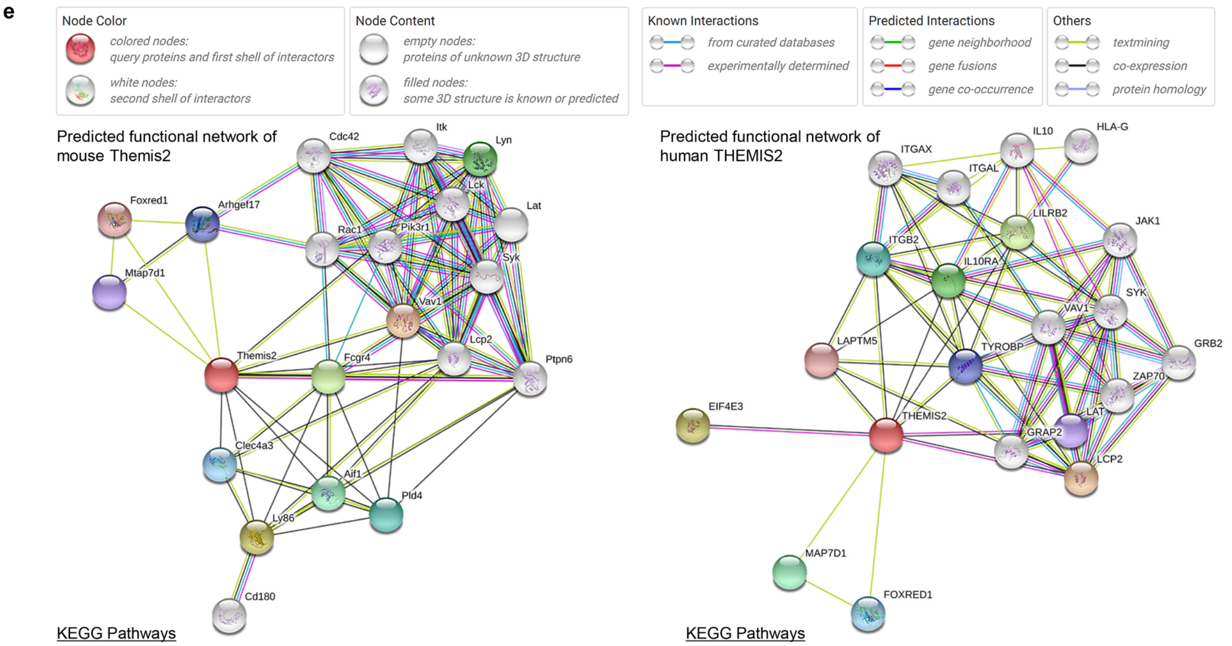

KEGG Pathways (mouse Themis2)

Pathway ID	Pathway description	FDR
04650	**Natural killer cell mediated cytotoxicity**	1.09e-13
04660	T cell receptor signaling pathway	4.1e-12
04664	Fc epsilon RI signaling pathway	1.88e-11
04666	Fc gamma R-mediated phagocytosis	6.41e-11
04662	B cell receptor signaling pathway	2.46e-9

KEGG Pathways (human THEMIS2)

Pathway ID	Pathway description	FDR
04650	**Natural killer cell mediated cytotoxicity**	4.07e-15
04660	T cell receptor signaling pathway	7.36e-10
04380	Osteoclast differentiation	2.01e-7
04664	Fc epsilon RI signaling pathway	4.2e-7
05152	Tuberculosis	8.47e-7

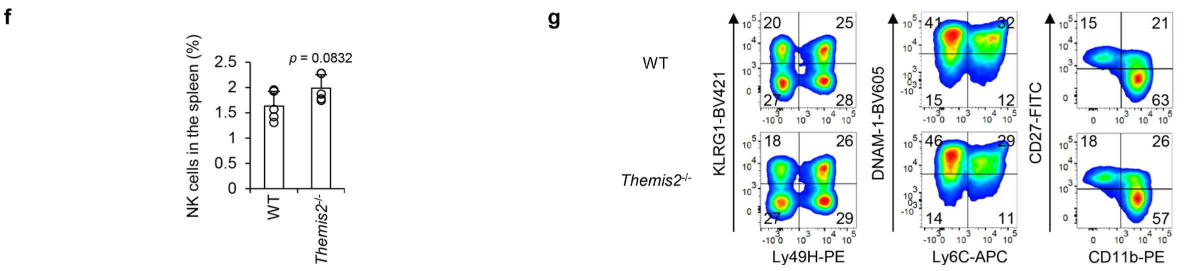

Supplementary Fig. 2. Identification of Themis2 as a candidate critical regulator of memory NK cell differentiation.

(**a**) Expression of Themis2 in natural killer (NK) cells (NK1.1^{+}CD3ε^{-}B220^{-} lymphocytes), T cells (NK1.1^{-}CD3ε^{+}B220^{-}CD11b^{-} lymphocytes), NKT cells (NK1.1^{+}CD3ε^{+}B220^{-}CD11b^{-} lymphocytes), B cells (NK1.1^{-}CD3ε^{-}B220^{+}CD11b^{-} lymphocytes), inflammatory monocytes (NK1.1^{-}CD3ε^{-}B220^{-}CD11b^{+}Ly6C^{+}Ly6G^{-} cells), and neutrophils (NK1.1^{-}CD3ε^{-}B220^{-}CD11b^{+}Ly6C$^{-\text{ to low}}$Ly6G^{+} cells) in the spleen of WT and *Themis2*$^{-/-}$ mice. Flow cytometry histograms are representative of 3 experiments (*n* = 1 mouse in each group). Data are pooled from 3 experiments (*n* = 3 mice in each group). Expression of Themis2 protein is represented as delta mean fluorescence intensity (*Δ*MFI) (MFI WT NK cells - MFI *Themis2*$^{-/-}$). (**b**) *Themis2* mRNA expression in NK cell subsets relative to that in naïve NK cells, as determined by quantitative reverse transcription PCR (qRT-PCR). Data are representative of 2 experiments (*n* = 3 mice (Naïve and Effector) and 5 mice (Cytokine-activated and Memory)). (**c**) Expression of *Themis2* mRNA in NK cell subsets. Normalized expression of *Themis2* mRNA by RNA-seq is shown (*n* = 3 mice). (**d**) Expression of Themis2 in naïve Ly49H^{+} and Ly49H^{-} NK cells (yellow fluorescent protein (YFP)$^{+}$NK1.1^{+}TCRβ^{-}KLRG1^{+} lymphocytes), effector Ly49H^{+} and Ly49H^{-} NK cells (YFP^{+}NK1.1^{+}TCRβ^{-}KLRG1^{high} lymphocytes), long-lived memory NK cells (YFP^{+}NK1.1^{+}TCRβ^{-}Ly49H^{+}KLRG1^{high} lymphocytes), and long-lived cytokine-activated NK cells (YFP^{+}NK1.1^{+}TCRβ^{-}Ly49H^{-}KLRG1^{high} lymphocytes) in

the spleen of naïve and mouse cytomegalovirus (MCMV)-infected *Themis2*$^{+/+}$ and *Themis2*$^{-/-}$ NK-CreERT2 mice. Flow cytometry histograms are representative of 2 experiments (*n* = 2 mice (Naïve and Effector) and 3 mice (Cytokine-activated and Memory)). Data are pooled from 2 experiments (*n* = 5 mice (Naïve and Effector) and 6 mice (Cytokine-activated and Memory)). Expression of Themis2 protein is represented as *Δ*MFI (MFI *Themis2*$^{+/+}$ NK cells - MFI *Themis2*$^{-/-}$). (**e**) Functional protein association networks of mouse Themis2 and human THEMIS2 by STRING and KEGG Pathways implemented by these networks. (**f**) Percentages of NK cells in the spleen of WT and *Themis2*$^{-/-}$ mice. Data are pooled from 2 experiments (*n* = 5 mice in each group). (**g**) Phenotype of naïve NK cells in the spleen of WT and *Themis2*$^{-/-}$ mice. Flow cytometry plots are representative of 2 experiments (*n* = 3 in each group). Statistical analysis was performed using one-way ANOVA (**b**, **c**, and **d**) and two-sided Student's *t*-test (**f**). Data are presented as mean values +/- SD (**a**-**d** and **f**).

Supplementary Fig. 3

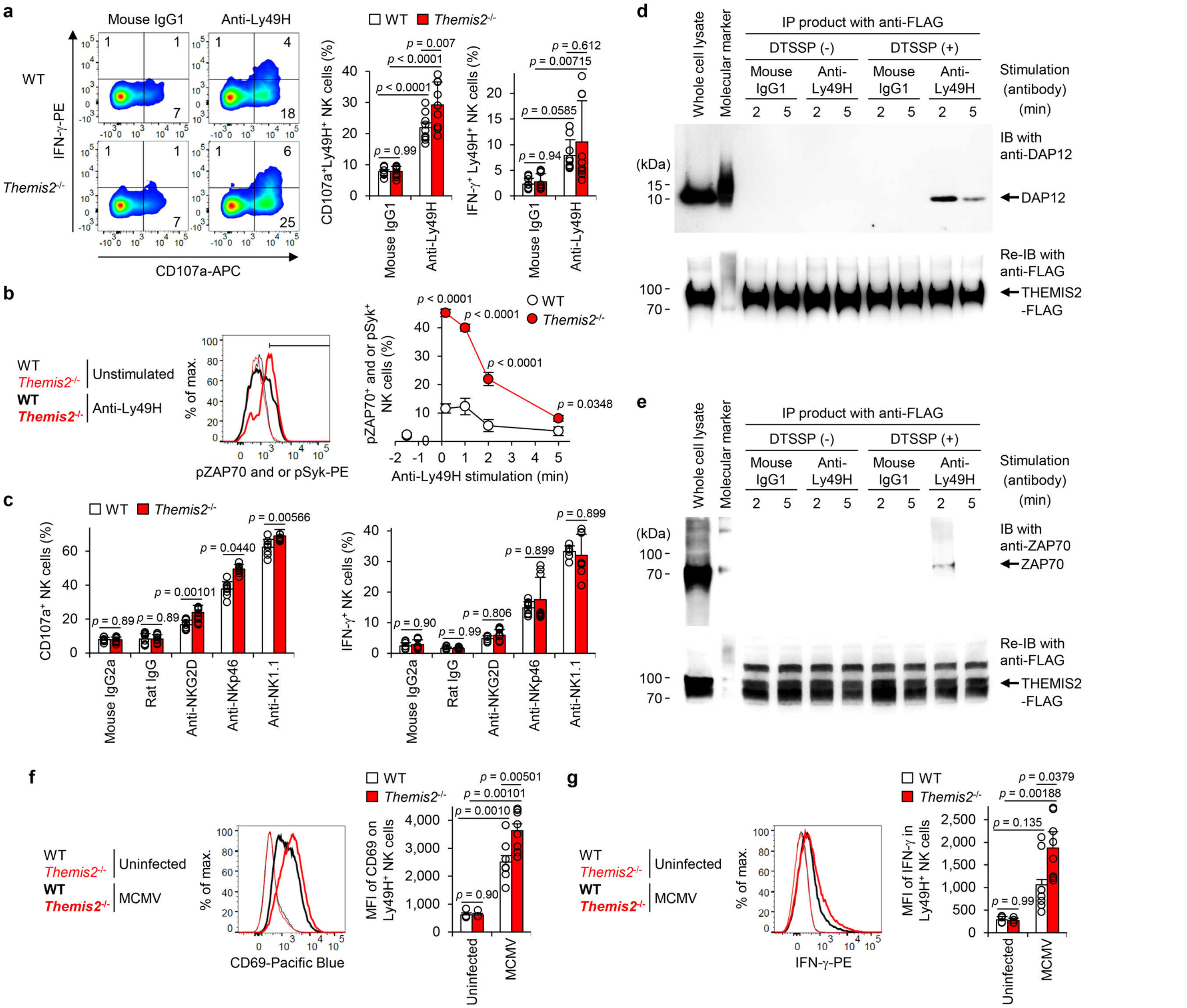

Supplementary Fig. 3. Themis2 inhibits NK cell activation via Ly49H.

(**a**) Degranulation and interferon-γ (IFN-γ) production of naïve WT and *Themis2*$^{-/-}$ Ly49H^{+} natural killer (NK) cells after stimulation with anti-Ly49H mAb. Flow cytometry plots are representative of 3 experiments (*n* = 3 wells in each group). Data are pooled from 3 experiments (*n* = 9 wells in

each group). (**b**) Phosphorylation of ZAP70 and Syk in naïve WT and *Themis2*$^{-/-}$ NK cells after Ly49H stimulation. Flow cytometry histograms are representative of 3 experiments (*n* = 3 wells in each group). Data are pooled from 3 experiments (*n* = 9 wells in each group). (**c**) Degranulation and IFN-γ production of naïve WT and *Themis2*$^{-/-}$ NK cells after stimulation with anti-NKG2D, anti-NKp46, and anti-NK1.1 mAbs. Data are pooled from 3 experiments (*n* = 8 wells (Anti-NK1.1) and 9 wells (Mouse IgG2a, Rat IgG, Anti-NKG2D, Anti-NKp46)). (**d** and **e**) Interaction of THEMIS2 and DAP12 (**d**) and ZAP70 (**e**) in the cytoplasm. NKL-Ly49H cells expressing THEMIS2-FLAG were stimulated with anti-Ly49H mAb for 2 or 5 min. Cytosolic proteins were treated with or without a chemical crosslinker DTSSP, THEMIS2-FLAG proteins were immunoprecipitated (IP) with anti-FLAG mAb, and immunoblotted (IB) with anti-DAP12 mAb (**d**) or anti-ZAP70 mAb (**e**), followed by reblotting with anti-FLAG mAb (**d** and **e**). The image is representative 3 experiments. (**f** and **g**) An activation marker CD69 (**f**) and IFN-γ production (**g**) of Ly49H^{+} NK cells in the spleen of uninfected or mouse cytomegalovirus (MCMV)-infected WT and *Themis2*$^{-/-}$ mice on day 1.5 pi. Flow cytometry histograms are representative of 2 experiments (*n* = 2 mice (Uninfected) and 4 mice (MCMV)). Expressions of CD69 and IFN-γ are represented as mean fluorescence intensity (MFI). Data are pooled from 2 experiments (*n* = 4 mice (Uninfected) and 8 mice (MCMV)). Statistical analysis was performed using one-way ANOVA (**a-c**, **f**, **g**). Data are presented as mean values +/- SD (**a-c**, **f**, and **g**).

Supplementary Fig. 4

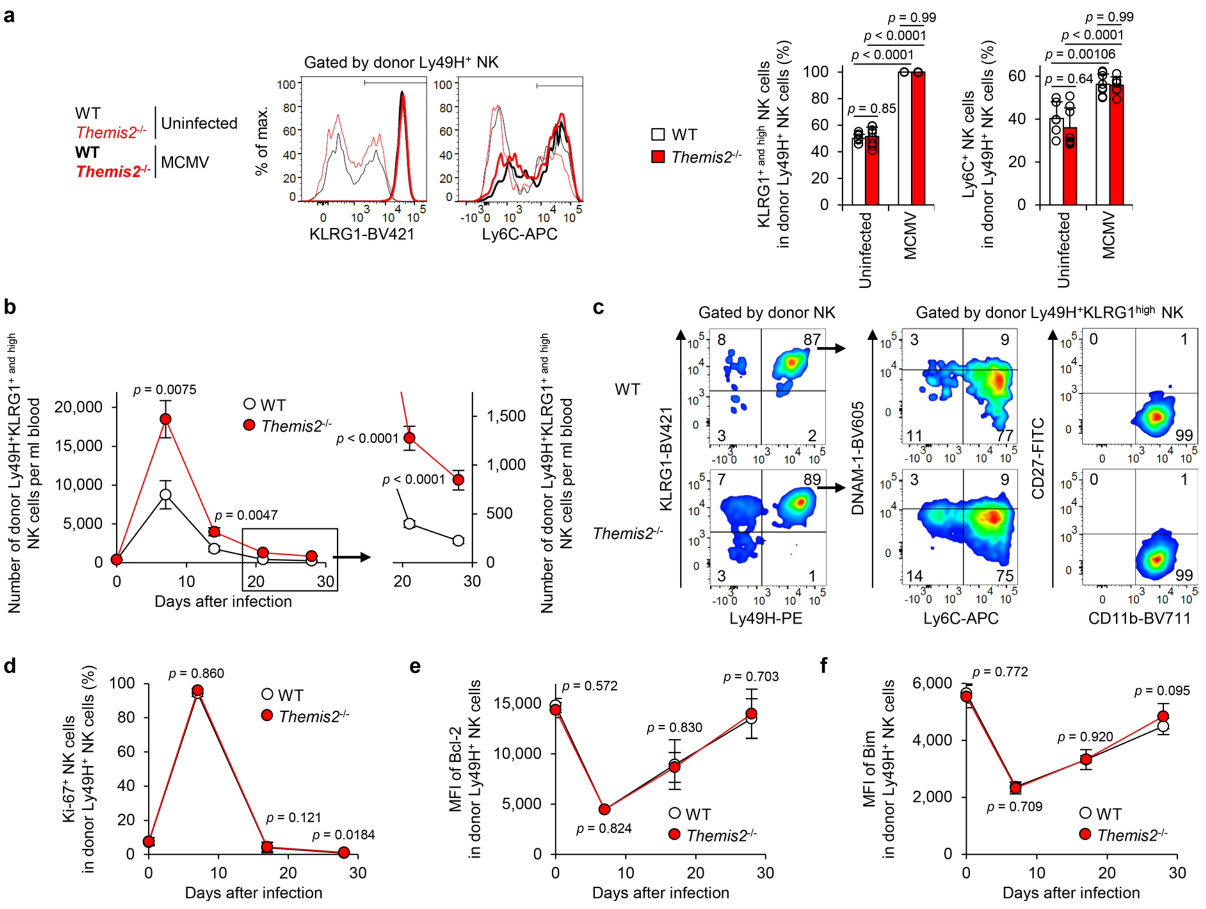

Supplementary Fig. 4. Themis2 inhibits memory NK cell differentiation.

(**a**) Expression of activation and differentiation markers KLRG1 and Ly6C on donor WT and *Themis2*$^{-/-}$ Ly49H^{+} natural killer (NK) cells in the blood on day 7 pi. Flow cytometry histograms are representative of 2 experiments (*n* = 4 mice in each group). Data are pooled from 2 experiments (*n* = 8 mice in each group). (**b**) Number of donor WT and *Themis2*$^{-/-}$ Ly49H^{+}KLRG1$^{+\text{ and high}}$ NK cells in the blood during mouse cytomegalovirus (MCMV) infection. Data are pooled from 2 experiments (*n* = 8 mice in each group for KLRG1$^{+\text{ to high}}$ NK cells and *n* = 6 mice

(Uninfected WT), 7 mice (MCMV *Themis2*$^{-/-}$), and 8 mice (Uninfected *Themis2*$^{-/-}$ and MCMV WT) for Ly6C^{+} NK cells). (**c**) Phenotype of donor WT and *Themis2*$^{-/-}$ NK cells in the spleen on day 28 pi. Flow cytometry plots are representative of 2 experiments (*n* = 4 mice in each group). (**d**-**f**) Expression of Ki-67 (**d**), Bcl-2 (**e**), and Bim (**f**) in donor WT and *Themis2*$^{-/-}$ Ly49H^{+}KLRG1$^{+\text{ and high}}$ NK cells in the spleen during MCMV infection. Expressions of Bcl-2 and Bim are represented as mean fluorescence intensity (MFI). Data are pooled from 2 experiments (*n* = 3 mice (day 0), 6 mice (days 7 and 17 pi), and 8 mice (day 28 pi)). Statistical analysis was performed using one-way ANOVA (**a**, **b**, and **d**-**f**). Data are presented as mean values +/- SD (**a**, **b**, and **d**-**f**).

Supplementary Fig. 5

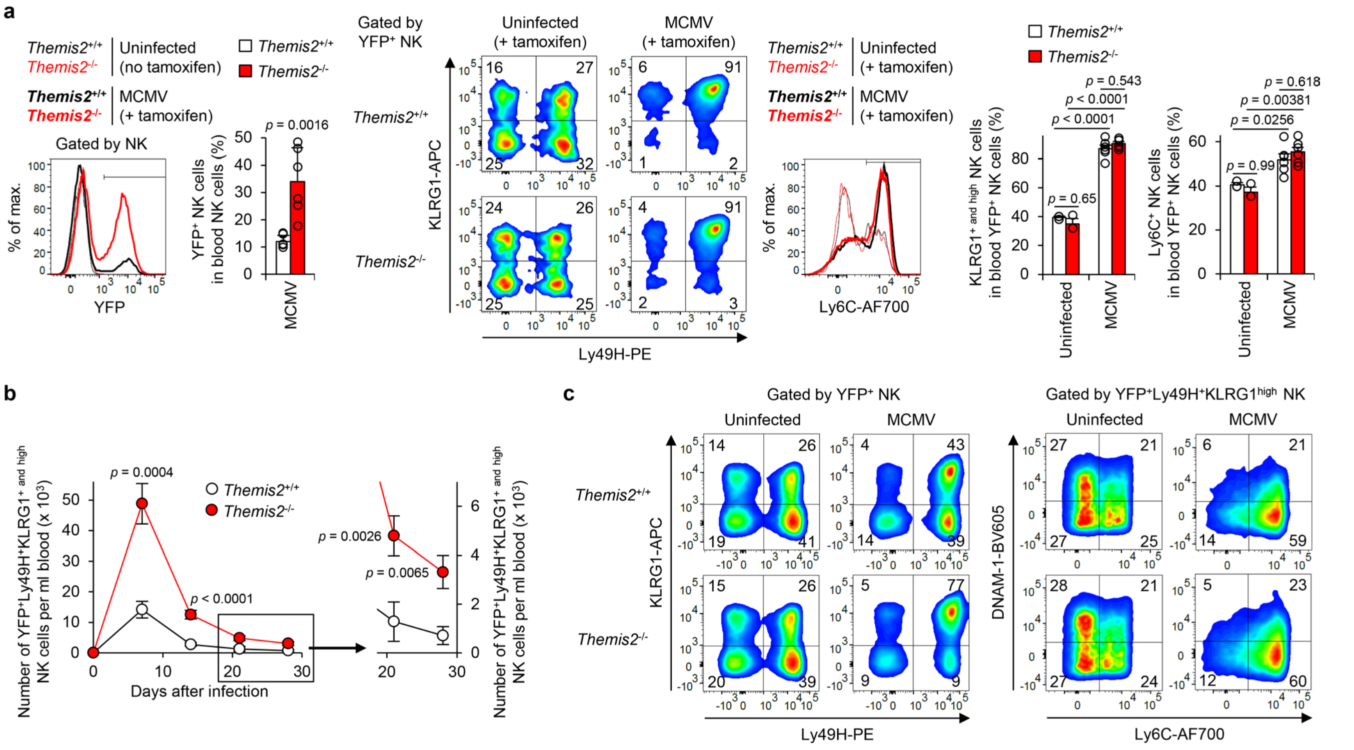

Supplementary Fig. 5. Themis2 inhibits memory NK cell differentiation.

(**a**) Phenotype of natural killer (NK) cells in the blood of uninfected and mouse cytomegalovirus (MCMV)-infected *Themis2*$^{+/+}$ and *Themis2*$^{-/-}$ NK-CreERT2 mice. Expression of yellow fluorescent protein (YFP) in NK cells on day 7 pi and expressions of the activation and differentiation markers KLRG1 and Ly6C and Ly49H on YFP^{+} NK cells in the blood of uninfected and MCMV-infected mice on day 7 pi are shown. Flow cytometry histograms are representative of 2 experiments (n = 2 mice (Uninfected) and 3 mice (MCMV)). Data are pooled from 2 experiments (n = 3 mice (Uninfected) and 6 mice (MCMV)). (**b**) Number of *Themis2*$^{+/+}$ and *Themis2*$^{-/-}$ YFP^{+}Ly49H^{+}KLRG1$^{+\text{ and high}}$ NK cells in the blood during MCMV infection. Data are

pooled from 2 experiments (*n* = 6 mice in each group). (**c**) Phenotype of YFP^{+} NK cells in the spleen of naïve and MCMV-infected *Themis2*$^{+/+}$ and *Themis2*$^{-/-}$ NK-CreERT2 mice on day 28 pi. Expression of Ly49H and KLRG1 on YFP^{+} NK cells and expression of Ly6C and DNAM-1 on $YFP^{+}Ly49H^{+}KLRG1^{+\text{ and high}}$ NK cells in naïve and MCMV-infected mice on day 28 pi are shown. Flow cytometry plots are representative of 2 experiments (*n* = 3 mice in each group). Statistical analysis was performed using two-sided Student's *t*-test (% YFP^{+} NK cells in **a**) and one-way ANOVA ($KLRG1^{+\text{ and high}}$ and $Ly6C^{+}$ NK cells in **a** and **b**). Data are presented as mean values +/- SD (**a** and **b**).

Supplementary Fig. 6

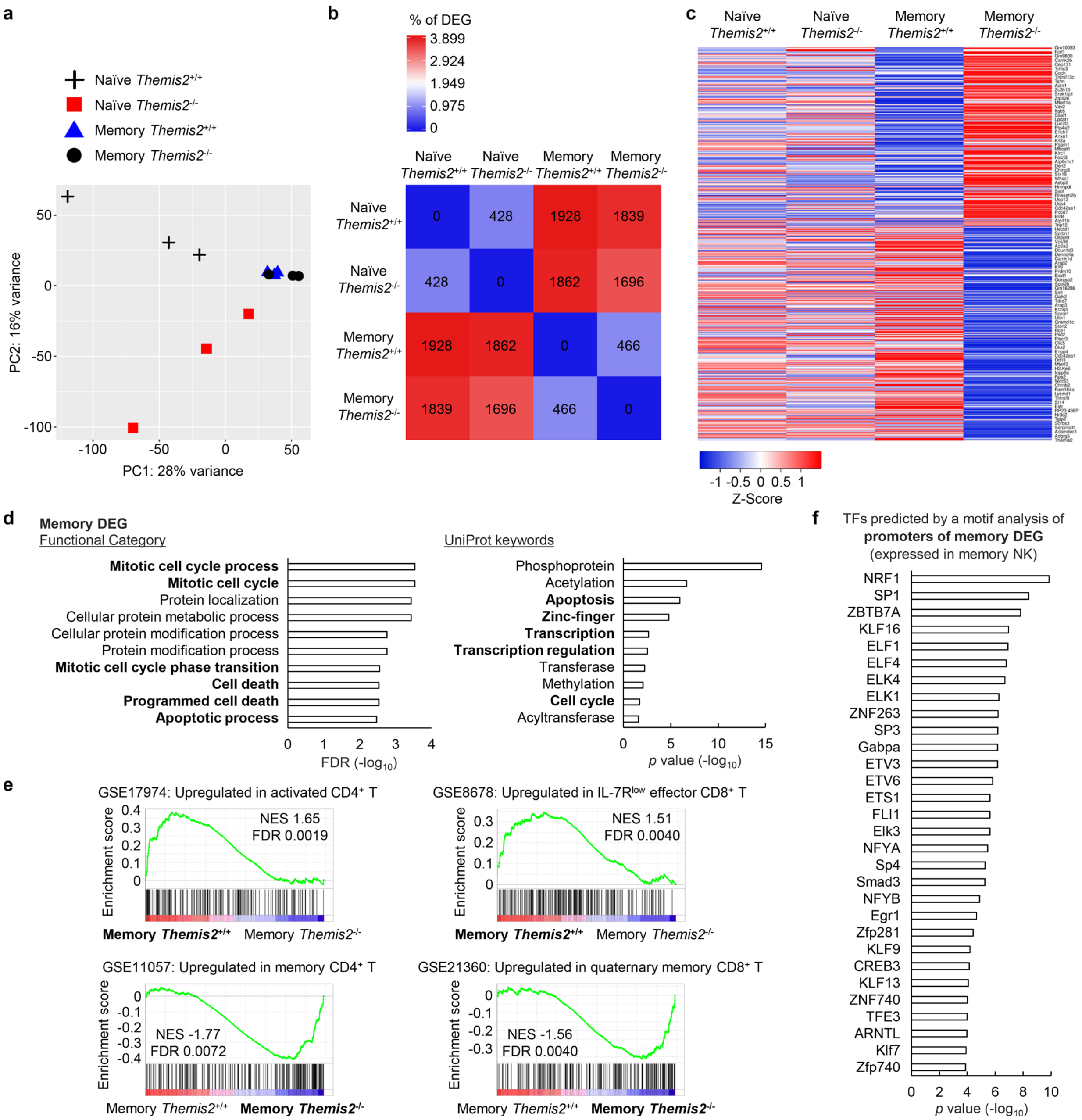

Supplementary Fig. 6. Themis2 regulates transcriptional changes in memory NK cells.

RNA-seq analysis of naïve *Themis2*$^{+/+}$ and *Themis2*$^{-/-}$ Ly49H^{+} natural killer (NK) cells and memory *Themis2*$^{+/+}$ and *Themis2*$^{-/-}$ NK cells (*n* = 3 mice in each group). (**a**) Principal component

analysis (PCA) of naïve *Themis2*$^{+/+}$ and *Themis2*$^{-/-}$ Ly49H^{+} NK cells and memory *Themis2*$^{+/+}$ and *Themis2*$^{-/-}$ NK cells. (**b**) Pairwise heatmap of differentially expressed genes (DEG). Percentages of DEGs are represented as the color-coded heatmap and the number of DEGs in each module is shown. (**c**) Heatmap of memory DEGs with Z-score. (**d**) Gene ontology (GO) analysis of memory DEGs. GO terms of Functional Category and UniProt keywords are shown with false discovery rate (FDR) and *p* values, respectively. (**e**) Gene set enrichment analysis (GSEA) of memory *Themis2*$^{+/+}$ and *Themis2*$^{-/-}$ NK cells. Enrichment of gene sets are shown with NES and FDR. (**f**) Transcription factors (TFs) that can bind to enriched motifs in memory DEG promoters. Transcription factors expressed in memory NK cells were ranked by *p* values.

Supplementary Fig. 7

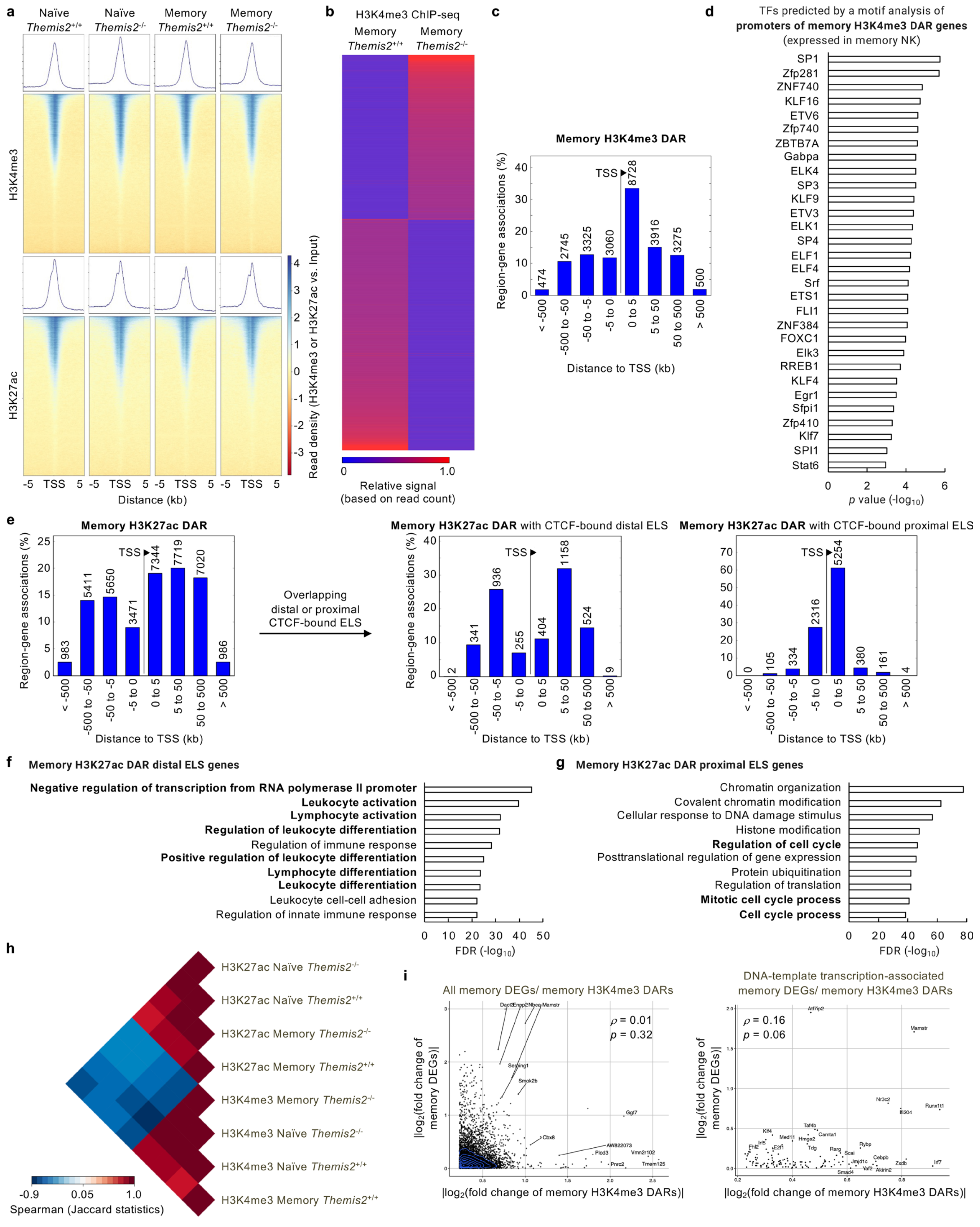

Supplementary Fig. 7. Themis2 regulates epigenetic changes in memory NK cells.

Tri-methylation at the 4th lysine residue of the histone H3 (H3K4me3) and acetylation at the 27th lysine residue of histone H3 (H3K27ac) ChIP-seq analysis of naïve *Themis2*$^{+/+}$ and *Themis2*$^{-/-}$ Ly49H^{+} natural killer (NK) cells and memory *Themis2*$^{+/+}$ and *Themis2*$^{-/-}$ NK cells (n = 3 mice in each group). (**a**) Epigenome-wide analysis of H3K4me3 and H3K27ac in naïve and memory *Themis2*$^{+/+}$ and *Themis2*$^{-/-}$ NK cells. The read density of H3K4me3 and H3K27ac in 5 kb of transcription start sites (TSS) is shown. (**b**) Heatmap of differentially accessible H3K4me3 regions between memory *Themis2*$^{+/+}$ and *Themis2*$^{-/-}$ NK cells (memory H3K4me3 differentially accessible regions (DAR)) with the relative read count. (**c**) Distance from memory H3K4me3 DARs to TSS with the number of DARs within each distance range. (**d**) Transcription factors (TFs) that can bind to enriched motifs in memory H3K4me3 DAR gene promoters. Transcription factors expressed in memory NK cells were ranked by p values. (**e**) Distance from differentially accessible H3K27ac regions between memory *Themis2*$^{+/+}$ and *Themis2*$^{-/-}$ NK cells (memory H3K27ac DARs) and memory H3K27ac DARs with CTCF-bound distal and proximal ELS to TSS with the number of DARs within each distance range. (**f** and **g**) Gene ontology (GO) analysis of memory H3K27ac DAR genes. GO terms of Biological Process of memory H3K27ac DAR distal ELS genes (**f**) and memory H3K27ac DAR proximal ELS genes (**g**) are shown with false discovery rate (FDR). (**h**) Pairwise intersections of the unified atlas of H3K4me3 and H3K27ac

in naïve and memory *Themis2*$^{+/+}$ and *Themis2*$^{-/-}$ NK cells. The pairwise intersection heatmap is shown with Spearman's correlation values. (**i**) Scatter plots depicting the fold change of normalized expression of memory differentially expressed genes (DEG) and the fold change of signal per million reads of memory H3K4me3 DARs. Spearman's correlation coefficients of all and DNA-templated transcription-associated memory DEGs/ memory H3K4me3 DARs with *p* values are shown.

Supplementary Fig. 8

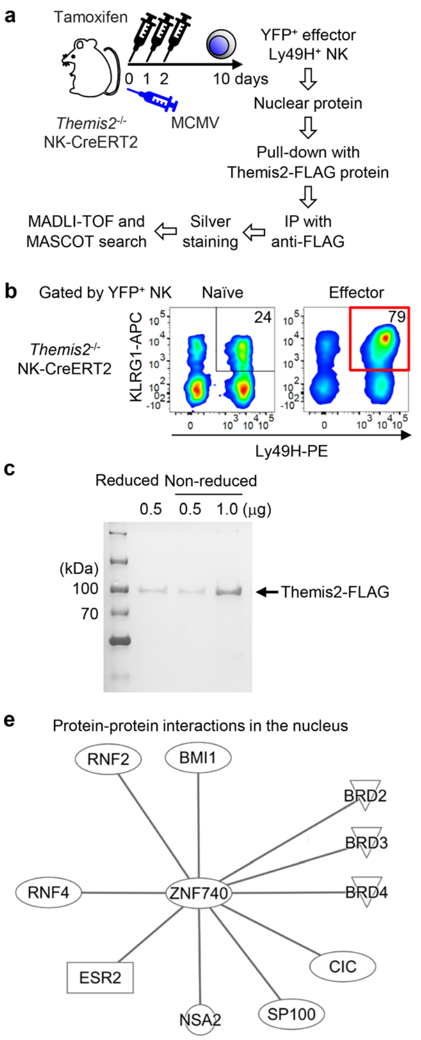

d

Criteria for a binding partner of Themis2 in the nucleus

1. Manually picking up unique peaks in test gel pieces vs. blank gel pieces and reagents alone by MALDI-TOF
2. MASCOT search ($p < 0.05$): 35 proteins
3. Transcription factors and epigenetic enzymes by UniProt: 15 transcription factors and 1 epigenetic enzyme
4. High expression in NK cells by RNA-seq: 9 transcription factors and 1 epigenetic enzyme

Protein	Gene	*Score	Description
cAMP-dependent transcription factor ATF-1	Atf1	90	Transcription factor
cAMP-responsive element modulator	Crem	84	Transcriptional activator or repressor
THAP domain-containing protein 11	Thap11	82	Transcriptional repressor
D site-binding protein	Dbp	63	Transcriptional activator
Homeobox protein Hox-B4	Hoxb4	59	Transcription factor
cAMP-responsive element binding protein 1	Creb1	54	Transcription factor
Zinc finger protein 622	Zfp622	49	Transcriptional activator?
Zinc finger protein 740	Zfp740	38	Zinc finger TF with unknown function
Mediator of RNA pol II transcription subunit 3	Med30	27	Transcriptional coactivator
Isoform 2 of Protein SET	Set	19	HAT inhibitor

*Score: -10 x $\log_{10}(p$ value) vs. Random

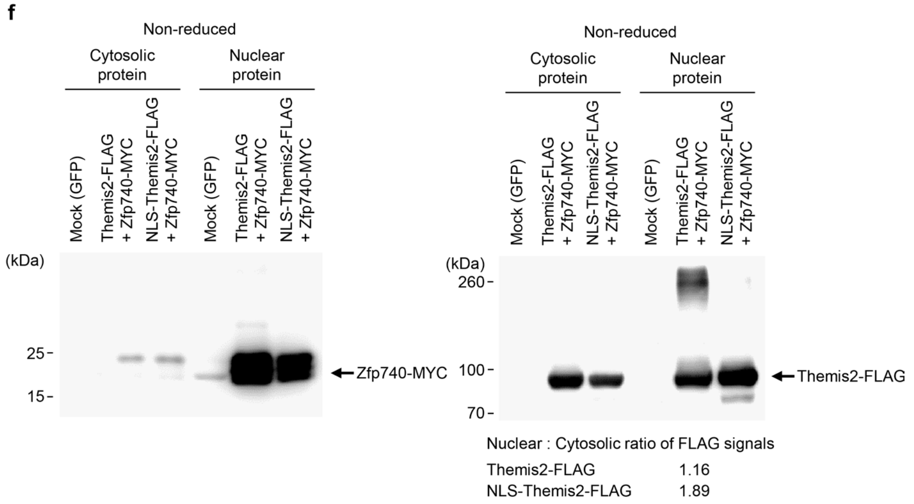

Supplementary Fig. 8. Identification and characterization of Zfp740.

(**a**) Schematic representation of the strategy used to identify Themis2-binding transcription factors. Nuclear proteins of effector *Themis2*$^{-/-}$ natural killer (NK) cells were incubated with Themis2-FLAG, and Themis2-bound proteins were immunoprecipitated. Unique protein bands of the immunoprecipitated (IP) products were analyzed by mass spectrometry. (**b**) Effector *Themis2*$^{-/-}$ yellow fluorescent protein (YFP)$^{+}$Ly49H^{+}KLRG1^{high} NK cells for nuclear proteins. The

red box represents the gating of effector NK cells. Flow cytometry plots are representative of 5 experiments (*n* = 1 mouse (Naïve) and 6 mice (Effector) in each experiment). (**c**) Quality control of Themis2-FLAG. The purity and the quality (e.g. monomer, aggregation) were confirmed by silver staining. The image is representative of 11 experiments. (**d**) Mass spectrometric analysis of Themis2-binding nuclear proteins. Proteins detected by the mass spectrometry analysis and a MASCOT search were narrowed down by the indicated criteria. Transcription factors and epigenetic enzymes are listed with MASCOT search scores. (**e**) Ingenuity Pathway Analysis of human ZNF740. Protein-protein interactions of human ZNF740 in the nucleus are shown. (**f**) Subcellular localization of Themis2 and Zfp740. Cytosolic and nuclear proteins of 293T cells co-expressing Zfp740-MYC and either Themis2-FLAG or NLS-Themis2-FLAG were immunoblotted with anti-MYC-tag mAb, followed by reblotting with anti-FLAG mAb. The nuclear translocation efficiency of Themis2-FLAG and NLS-Themis2-FLAG was compared. These images are representative of 3 experiments.

Supplementary Fig. 9

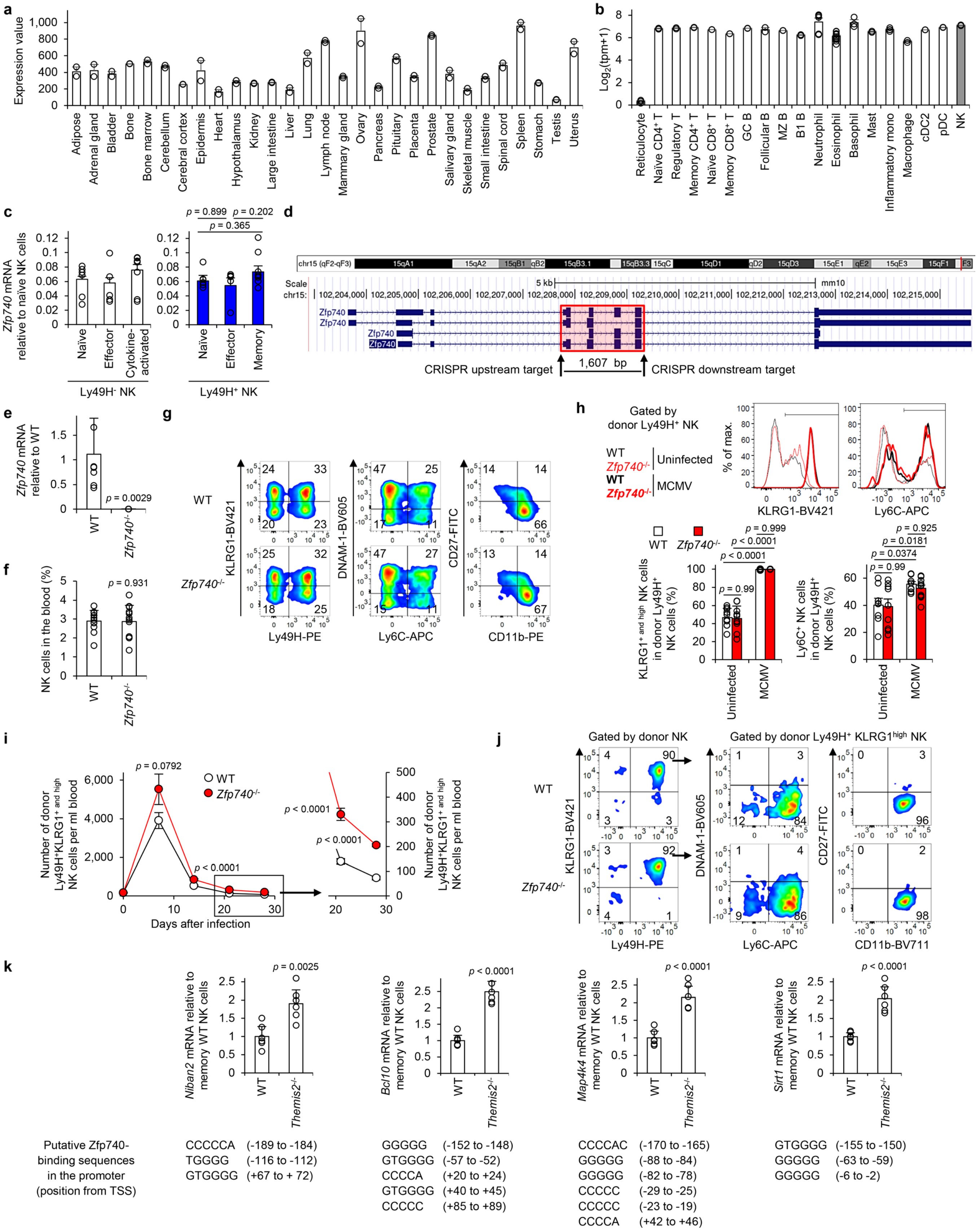

Supplementary Fig. 9. Phenotype of *Zfp740*$^{-/-}$ NK cells.

(**a** and **b**) Expression of *Zfp740* mRNA in mouse organs (*n* = 2 mice) (**a**) and in mouse immune cells (*n* = 2 samples (Naïve CD4^{+} T, Regulatory T, Memory CD4^{+} T, Naïve CD8^{+} T, Memory CD8^{+} T, GC B, Follicular B, MZ B, B1 B, Mast, Macrophage, cDC2, pDC, NK), 3 samples (Reticulocyte, Basophil), 4 samples (Inflammatory mono), 6 samples (Neutrophil), and 14 samples (Eosinophil)) (**b**). (**c**) *Zfp740* mRNA in natural killer (NK) cell subsets relative to naïve NK cells was analyzed by quantitative reverse transcription PCR (qRT-PCR). Data are pooled from 2 experiments (*n* = 6 mice (Naïve and Effector), 8 mice (Memory), and 9 mice (Cytokine-activated)). (**d**) Schematic representation of the strategy used to generate *Zfp740*$^{-/-}$ mice. (**e**) Expression of *Zfp740* mRNA in blood leukocytes in WT and *Zfp740*$^{-/-}$ mice by qRT-PCR. Data are pooled from 2 experiments (*n* = 7 mice (WT) and 9 mice (*Zfp740*$^{-/-}$)). (**f**) Percentages of NK cells in the blood of WT and *Zfp740*$^{-/-}$ mice. Data are pooled from 2 experiments (*n* = 12 mice in each group). (**g**) Phenotype of naïve NK cells in the blood of WT and *Zfp740*$^{-/-}$ mice. Flow cytometry plots are representative of 2 experiments (*n* = 6 mice in each group). (**h**-**j**) Recipient *Tyrobp*$^{-/-}$ mice received donor CD45.1^{+} WT Ly49H^{+} NK cells and CD45.2^{+} *Zfp740*$^{-/-}$ Ly49H^{+} NK cells at a 1:1 ratio and then infected with mouse cytomegalovirus (MCMV). (**h**) Expressions of KLRG1 and Ly6C on WT and *Zfp740*$^{-/-}$ Ly49H^{+} NK cells in the blood on day 7 pi. Flow cytometry histograms

are representative of 2 experiments (*n* = 4 mice (Uninfected) and 6 mice (MCMV) in each group). Data are pooled from 2 experiments (*n* = 10 mice in each group for KLRG1$^{+\text{ to high}}$ NK cells and *n* = 8 mice (Uninfected WT), 9 mice (Uninfected *Themis2*$^{-/-}$), and 10 mice (MCMV) for Ly6C^{+} NK cells). (**i**) Number of WT and *Zfp740*$^{-/-}$ Ly49H^{+}KLRG1$^{+\text{ and high}}$ NK cells in the blood during MCMV infection. Data are pooled from 2 experiments (*n* = 10 mice in each group). (**j**) Phenotype of WT and *Zfp740*$^{-/-}$ NK cells in the spleen on day 28 pi. Flow cytometry plots are representative of 2 experiments (*n* = 6 mice in each group). (**k**) Expression of mRNA of anti-apoptosis-associated genes with promoters bearing putative Zfp740-binding sequences in memory *Themis2*$^{-/-}$ NK cells relative to memory WT NK cells was analyzed by qRT-PCR. Relative expression, putative Zfp740-binding sequences, and their positions in the promoters from their transcription start sites (TSS) are shown. Data are pooled from 2 experiments (*n* = 6 mice in each group). Statistical analysis was performed using one-way ANOVA (**c**, **h**, and **i**) and two-sided Student's *t*-test (**e**, **f**, and **k**). Data are presented as mean values +/- SD (**a**-**c**, **e**-**f**, **h**-**i**, and **k**).

Supplementary Fig. 10

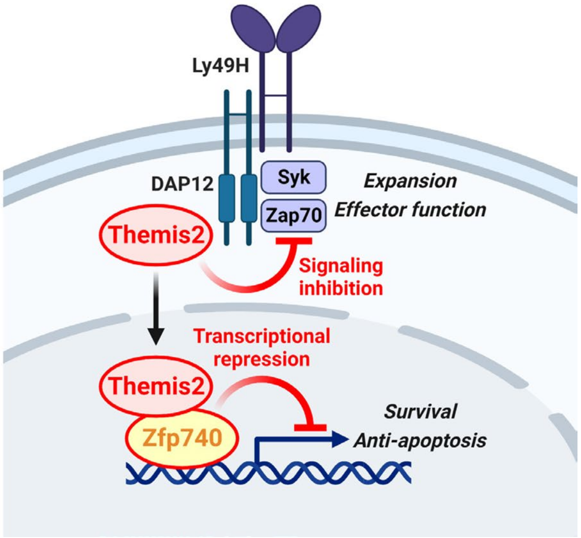

Supplementary Fig. 10. Themis2 limits NK memory formation and function.

Themis2 inhibits activation signaling via Ly49H by attenuating the activation of ZAP70 and/or Syk in the cytoplasm, which restricts effector function and expansion of memory Ly49H$^+$ natural killer (NK) cells. Themis2 was translocated into the nucleus in Ly49H$^+$ NK cells following mouse cytomegalovirus (MCMV) infection. Themis2 promotes Zfp740-mediated transcriptional repression of anti-apoptosis-associated genes to regulate the persistence of memory NK cells. Therefore, Themis2 qualitatively and quantitatively limits the formation of NK cell memory.
