# Supplementary material for: Themis2 regulates natural killer cell memory function and formation
Source: Nat Commun. 2023 Nov 8;14:7200. doi: 10.1038/s41467-023-42578-8 (PMC10632368; doi:10.1038/s41467-023-42578-8)
Supplement: Supplementary file 3 — Reporting Summary [file 41467_2023_42578_MOESM3_ESM.pdf]

Corresponding author(s): Akria Shibiya
Tsukasa Nabekura

Last updated by author(s): Sep 26, 2023

Reporting Summary

Nature Portfolio wishes to improve the reproducibility of the work that we publish. This form provides structure for consistency and transparency in reporting. For further information on Nature Portfolio policies, see our Editorial Policies and the Editorial Policy Checklist.

Statistics

For all statistical analyses, confirm that the following items are present in the figure legend, table legend, main text, or Methods section.

n/a	Confirmed	
☐	☒	The exact sample size (*n*) for each experimental group/condition, given as a discrete number and unit of measurement
☐	☒	A statement on whether measurements were taken from distinct samples or whether the same sample was measured repeatedly
☐	☒	The statistical test(s) used AND whether they are one- or two-sided<br>*Only common tests should be described solely by name; describe more complex techniques in the Methods section.*
☐	☒	A description of all covariates tested
☐	☒	A description of any assumptions or corrections, such as tests of normality and adjustment for multiple comparisons
☐	☒	A full description of the statistical parameters including central tendency (e.g. means) or other basic estimates (e.g. regression coefficient) AND variation (e.g. standard deviation) or associated estimates of uncertainty (e.g. confidence intervals)
☐	☒	For null hypothesis testing, the test statistic (e.g. *F*, *t*, *r*) with confidence intervals, effect sizes, degrees of freedom and *P* value noted<br>*Give P values as exact values whenever suitable.*
☒	☐	For Bayesian analysis, information on the choice of priors and Markov chain Monte Carlo settings
☒	☐	For hierarchical and complex designs, identification of the appropriate level for tests and full reporting of outcomes
☒	☐	Estimates of effect sizes (e.g. Cohen's *d*, Pearson's *r*), indicating how they were calculated

Our web collection on statistics for biologists contains articles on many of the points above.

Software and code

Policy information about availability of computer code

Data collection

Flow cytometry: BD FACSDiva V8, FlowJo V10
Confocal laser microscopy: FLUOVIEW Ver4.1
RNA-seq and ChIP-seq: CLC Genomics Workbench 12.0, Heatmapper 1.0.0, MACS3 callpeak 3.0.0a6, Morpheus, Galaxy v21.09
Bioinformatics: DAVID v2021q4, Galaxy v21.09, GREAT v4.0.4, GSEA v4.2.3, Ingenuity Pathway Analysis 20.0, ShinyGO v0.61, STRING v10.5, Venny 2.0, iDEP.96
Motif analysis: MEME Suite 5.3.0
Mass spectrometry: flexControl 3.4, MASCOT 2.7
Biochemistry: ImageQuant TL ver8
Luciferase reporter assay: SkanIt RE 6.1.1
qPCR and qRT-PCR: 7500 software v2.0.6

Data analysis

Flow cytometry: Samples were run on LSRFortessa and FACSAria III (BD Biosciences) and the data were analyzed with FlowJo (FlowJo). The positivity, mean fluorescence Intensity (MFI), and delta MFI were calculated by FlowJo.
Confocal laser microscopy: Samples were observed under a FluoView FV10i (Olympus) and analyzed by FLUOVIEW (Olympus).
RNA-seq: RNA was isolated using a TRIzol reagent (Thermo Fisher Scientific), the yield was monitored using an Agilent Bioanalyzer RNA 6000 Pico Kit (Agilent Technologies), and processed to sequencing library using a SMART-seq Stranded Kit (Takara Bio). Sequencing was performed by NextSeq 500 (Illumina). FASTQ files were imported to CLC Genomics Workbench (Qiagen) and mapped to mm10. Normalized expression values were obtained by quantile normalization for the total count. DEGs were defined as having normalized expression > 10 and log2 (fold change) > 1 with FDR < 0.05. The heatmap of memory DEGs with relative expression with Z-score was drawn by using Heatmapper (http://www.heatmapper.ca/).
ChIP-seq: Cells were fixed with 1% formaldehyde (Sigma-Aldrich). ChIP assays were performed using anti-H3K4me3 Ab (Abcam) and anti-

H3K27ac Ab (Abcam). ChIP DNA was processed into a DNA sequencing library using NEBNext Ultra II DNA Library Prep Kit for Illumina (New England Biolabs) and paired-end sequencing was performed with NextSeq 500 (Illumina). Unique peaks versus input controls were identified using MACS3 (https://github.com/macs3-project/MACS) and the code is available in the GitHub repository (https://github.com/CAB314/Themis2-Nabekura). H3K4me3 DARs were extracted by bedtools on Galaxy (https://usegalaxy.org/) and these unique H3K4me3 DAR-associated single nearest genes were identified by using GREAT (http://great.stanford.edu/public/html/). H3K27ac DARs were extracted by Galaxy and then H3K27ac DARs with enhancer-like signatures (ELS) were identified by using CTCF-bound enhancer signatures. These unique H3K27ac DAR-associated single nearest genes with distal and proximal ELS were identified by using GREAT. The heatmap was drawn by Morpheus (https://software.broadinstitute.org/morpheus/). These epigenome-wide heatmaps of the read density in 5 kb from TSS were drawn by deepTools on Galaxy. The heatmap of pairwise intersections was drawn with their Spearman correlations for pairwise Jaccard statistics by deepTools on Galaxy. The distance from individual memory DARs to TSS was plotted with the number of DARs within each distance range by GREAT. An integrative analysis of memory DEGs and memory H3K4me3 DARs was performed by using the code available in the GitHub repository (https://github.com/CAB314/Themis2-Nabekura).
Bioinformatics: GO analysis was performed by DAVID (https://david.ncifcrf.gov/) and ShinyGO (http://bioinformatics.sdstate.edu/go/). GO analysis of DAR genes were performed by GREAT. Functional protein association networks were constructed and KEGG Pathways of their networks were implemented by STRING (https://string-db.org/). GSEA was performed and normalized enrichment score (NES) and FDR were computed by GSEA software (https://www.gsea-msigdb.org/gsea/index.jsp). The knowledge-based network analysis was performed by Ingenuity Pathway Analysis (Qiagen). The PCA was performed by iDEP (http://bioinformatics.sdstate.edu/idep96/).
Motif analysis: Transcription factor binding motifs in promoters of DEGs and H3K4me DAR genes were analyzed by the MEME Suite (https://meme-suite.org/meme/index.html). Enriched motifs in promoter sequences (200 bp upstream and 100 bp downstream of TSS) were discovered by MEME STREME. Transcription factors that can bind to these motifs were ranked by MEME Tomtom.
Mass spectrometry: Samples were were mixed with a-Cyano-4-hydroxycinnamic acid as a matrix substance (Shimadzu GLC), spotted onto a steel target plate, and analyzed by MALDI-TOF/MS with a ultrafleXtreme MALDI-TOF/TOF NTA and flexControl (Bruker). The resulting peptide peaks were manually picked up and proteins were identified through peptide mass fingerprint matching by MASCOT search (https://www.matrixscience.com/) with SWISS-PROT database (https://www.uniprot.org/) and the score (-10 x log10(p value) vs. random).
Biochemistry: The chemiluminescence was detected with ImageQuant LAS 4000 mini (Cytiva) and analyzed with ImageQuant TL ver8 (Cytiva).
Luciferase reporter assay: The luciferase-mediated luminescence and GFP fluorescence were measured with Varioskan LUX (Thermo Fisher Scientific) and analyzed with Skanlt (Thermo Fisher Scientific).
qPCR and qRT-PCR: qPCR and qRT-PCR were performed on an ABI 7500 Fast real-time PCR system (Thermo Fisher Scientific) and analyzed by 7500 software (Thermo Fisher Scientific).
Statistical analysis: The two-tailed Student's t-test and one-way ANOVA were used to compare the data by using GraphPad Prism 5 (GraphPad Software) or Quick Calcs of GraphPad Prism Resources (https://www.graphpad.com/resources).

For manuscripts utilizing custom algorithms or software that are central to the research but not yet described in published literature, software must be made available to editors and reviewers. We strongly encourage code deposition in a community repository (e.g. GitHub). See the Nature Portfolio guidelines for submitting code & software for further information.

Data

Policy information about availability of data

All manuscripts must include a data availability statement. This statement should provide the following information, where applicable:

- Accession codes, unique identifiers, or web links for publicly available datasets
- A description of any restrictions on data availability
- For clinical datasets or third party data, please ensure that the statement adheres to our policy

RNA-seq data, ChIP-seq data, and all the datasets generated in this study are available in GEO with accession number SuperSeries GSE227139 (GSE227085, GSE227137, and GSE227138).
Gene expression profiles in mouse organs are available in GEO with accession number GSE10246.
Gene expression profiles in mouse hematopoietic cells are available in GEO with accession number GSE116177.

Research involving human participants, their data, or biological material

Policy information about studies with human participants or human data. See also policy information about sex, gender (identity/presentation), and sexual orientation and race, ethnicity and racism.

Reporting on sex and gender	A healthy volunteer (male, Asian, Japanese, 37 years old) voluntarily donated blood.
Reporting on race, ethnicity, or other socially relevant groupings	A healthy volunteer (male, Asian, Japanese, 37 years old) voluntarily donated blood.
Population characteristics	Male, Asian, Japanese, 37 years old
Recruitment	We recruited healthy volunteers as blood donors in Japan through the approved procedures by the ethics committee for medical sciences at the University of Tsukuba (approval number 234-2). No potential self-selection bias and other biases have been identified.
Ethics oversight	These procedures were approved by the ethics committee for medical sciences at the University of Tsukuba (approval number 234-2). All participants underwent an informed consent process.

Note that full information on the approval of the study protocol must also be provided in the manuscript.

Field-specific reporting

Please select the one below that is the best fit for your research. If you are not sure, read the appropriate sections before making your selection.

☒ Life sciences ☐ Behavioural & social sciences ☐ Ecological, evolutionary & environmental sciences

For a reference copy of the document with all sections, see nature.com/documents/nr-reporting-summary-flat.pdf

Life sciences study design

All studies must disclose on these points even when the disclosure is negative.

Sample size

Suitable sample sizes were determined based on our previous studies to ensure adequate reproducibility of results. Sample size for mouse experiments was at least 3 per group. In case sample sizes are smaller than 3, results were confirmed by multiple independent experiments.

1. Type 1 innate lymphoid cells protect mice from acute liver injury via interferon-g secretion for upregulating Bcl-xL expression in hepatocytes. Immunity, 2020;52:96-108. Tsukasa Nabekura, Luke Riggan, Andrew D. Hildreth, Timothy E. O'Sullivan, and Akira Shibuya
2. Tracking the fate of antigen-specific versus cytokine-activated natural killer cells after cytomegalovirus infection. J. Exp. Med., 2016;213:2745-58. Tsukasa Nabekura and Lewis L. Lanier
3. Activating receptors for self-MHC class I enhance effector functions and memory differentiation of NK cells during mouse cytomegalovirus infection. Immunity, 2016;45:74-82. Tsukasa Nabekura and Lewis L. Lanier

Data exclusions

None

Replication

Results were reproducible across multiple experiments. Experiments were repeated independently at least 2 times.

Randomization

Experimental groups in all experiments were not randomized, because we individually maintained wild-type and genetically modified mice and used their specimens and immune cells with appropriate labels. Mice, their specimens, and immune cells were used in an age-matched and gender-matched manner in all experiments.

Blinding

Experiments were not performed in a blinded manner. Blinding was not relevant to our study, since we studied and compared the property of known biomaterials.

Reporting for specific materials, systems and methods

We require information from authors about some types of materials, experimental systems and methods used in many studies. Here, indicate whether each material, system or method listed is relevant to your study. If you are not sure if a list item applies to your research, read the appropriate section before selecting a response.

Materials & experimental systems

n/a	Involved in the study	
☐	☒	Antibodies
☐	☒	Eukaryotic cell lines
☒	☐	Palaeontology and archaeology
☐	☒	Animals and other organisms
☒	☐	Clinical data
☒	☐	Dual use research of concern
☒	☐	Plants

Methods

n/a	Involved in the study	
☐	☒	ChIP-seq
☐	☒	Flow cytometry
☒	☐	MRI-based neuroimaging

Antibodies

Antibodies used

Alexa Fluor 488-conjugated anti-human/mouse/rat Bim (C34C5), Cell Signaling Technology, 94805
Alexa Fluor 488-conjugated anti-rabbit IgG, donkey polyclonal Ab, Thermo Fisher Scientific, A-21206
Alexa Fluor 488-conjugated rabbit IgG (DA1E), Cell Signaling Technology, 2975
Alexa Fluor 647-conjugated anti-human/mouse Ki-67 (B56), BD Biosciences, 558615
Alexa Fluor 647-conjugated anti-rabbit IgG, donkey polyclonal Ig (Poly4064), BioLegend, 406414
Alexa Fluor 647-conjugated mouse IgG1 (MOPC-21), BioLegend, 400155
Alexa Fluor 700-conjugated anti-mouse CD45.1 (A20), BioLegend, 110724
Alexa Fluor 700-conjugated anti-mouse Ly6C (HK1.4), BioLegend, 128024
Alexa Fluor 700-conjugated anti-mouse Ly6G (1A8), BD Biosciences, 561236
Alexa Fluor 700-conjugated anti-mouse NKp46 (29A1.4), BD Biosciences, 561169
Anti-FLAG BioM2, Sigma-Aldrich, F9291
Anti-FLAG M2 affinity gel, Sigma-Aldrich, A2220
Anti-Mouse IgG, HRP-Linked Whole Ab Sheep, Cytiva, NA931-1ML
APC-conjugated anti-mouse B220 (RA3-6B2), BioLegend, 103212
APC-conjugated anti-mouse CD45.2 (104), BioLegend, 109814

APC-conjugated anti-mouse CD49a (HMa1), BioLegend, 142606
APC-conjugated anti-mouse CD107a (1D4B), BioLegend, 121614
APC-conjugated anti-mouse KLRG1 (2F1/KLRG1), BioLegend, 138412
APC-conjugated anti-mouse Ly6C (HK1.4), BioLegend, 128016
Biotinylated anti-mouse CD4 (GK1.5), BioLegend, 100404
Biotinylated anti-mouse CD5 (53-7.3), BioLegend, 100604
Biotinylated anti-mouse CD8 (53-6.7), BioLegend, 100704
Biotinylated anti-mouse CD19 (6D5), BioLegend, 115504
Biotinylated anti-mouse DNAM-1 (TX42.1), made by our laboratory (Nabekura et al., 2014)
Biotinylated anti-mouse Gr-1 (RB6-8C5), BioLegend, 108404
Biotinylated anti-mouse Ly49H (3D10), Thermo Fisher Scientific, 13-5886-82
Biotinylated anti-mouse TCRb (H57-597), BioLegend, 109204
Biotinylated anti-mouse TER-119 (TER-119), BioLegend, 116204
Brilliant Violet 421-conjugated anti-mouse KLRG1 (2F1/KLRG1), BioLegend, 138414
Brilliant Violet 711-conjugated anti-mouse CD11b (M1/70), BioLegend, 101242
FITC-conjugated anti-human CD3 (HIT3a), BD Biosciences, 555339
FITC-conjugated anti-human CD19 (HIB19), BD Biosciences, 555412
FITC-conjugated anti-mouse CD11b (M1/70), BD Biosciences, 561688
FITC-conjugated anti-mouse CD27 (LG.3A10), BioLegend, 124208
FITC-conjugated anti-mouse CD45.2 (104), BioLegend, 109806
FITC-conjugated anti-mouse CD49b (DX5), BioLegend, 108906
FITC-conjugated anti-mouse TCRb (H57-597), BioLegend, 109206
HRP-conjugated anti-rabbit IgG (Poly4064), BioLegend, 406401
Pacific Blue-conjugated anti-mouse CD3e (145-2C11), BioLegend, 100334
Pacific Blue-conjugated anti-mouse CD69 (H1.2F3), BioLegend, 104524
PE-conjugated human/mouse ZAP70 (Y319) and or Syk (Y352) (17A/P-ZAP70), BD Biosciences, 557881
PE-conjugated anti-mouse Bcl-2 (BCL/10C4), BioLegend, 633508
PE-conjugated anti-human CD16 (3G8), BD Biosciences, 560995
PE-conjugated anti-mouse CD11b (M1/70), BioLegend, 101208
PE-conjugated anti-mouse IFN-g (XMG1.2), BioLegend, 505808
PE-conjugated anti-mouse Ly49H (3D10), BioLegend, 144706
PE-conjugated anti-rabbit IgG, donkey polyclonal Ig (Poly4064), BioLegend, 406421
PE-conjugated mouse IgG1 (MOPC-21), BD Biosciences, 559320
PECy7-conjugated anti-mouse B220 (RA3-6B2), BioLegend, 103222
PECy7-conjugated anti-mouse Ly6C (AL-21), BD Biosciences, 560593
PECy7-conjugated anti-mouse TCRb (H57-597), BioLegend, 109222
PerCPCy5.5-conjugated anti-mouse NK1.1 (PK136), BioLegend, 108728
Purified anti-human/mouse/rat THEMIS2, rabbit polyclonal IgG, ABclonal, A15355
Purified anti-DAP12 (D7G1X), Cell Signaling Technology, 12492
Purified anti-GFP (D5.1), rabbit mAb, Cell Signaling Technology, 2956
Purified anti-Histone H3 (acetyl K27) antibody - ChIP Grade, Abcam, ab4729
Purified anti-Histone H3 (tri methyl K4) antibody - ChIP Grade, Abcam, ab8580
Purified anti-human/mouse ICB1, rabbit polyclonal IgG, Abcam, ab236975
Purified anti-mouse CD16/32 (2.4G2), BD Biosciences, 553142
Purified anti-mouse Ly49H (3D10), BioLegend, 144702
Purified anti-mouse NK1.1 (PK136), ATCC, HB-191
Purified anti-mouse NKG2D (CX5), BioLegend, 130218
Purified anti-mouse NKp46 (29A1.4), BioLegend, 137640
Purified anti-Myc-tag (9B11), Cell Signaling Technology, 2276
Purified anti-ZAP70 (99F2), Cell Signaling Technology, 2705
Purified mouse IgG1 (MOPC-21), BioLegend, 400197
Purified mouse IgG2a (MOPC-173), BioLegend, 400281
Purified polyclonal mouse IgG, Bio X Cell, BE0093
Purified rat IgG1 (R3-34), BD Biosciences, 553921
Purified rat IgG2a (R35-95), BD Biosciences, 555840
V450-conjugated human CD56 (B159), BD Biosciences, 560360
The dilution factors for these antibodies for flow cytometry were 50 to 1000, or these antibodies were diluted and used according to the manufacturers' instructions.
The dilution factors for these antibodies for biochemistry were 1000 to 2000, or these antibodies were diluted and used according to the manufacturers' instructions.
For immunofluorescence staining, dilution factors for anti-GFP rabbit mAb (D5.1) and AF488-conjugated anti-rabbit IgG were 75 and 1000, respectively. Amounts of rabbit anti-human/mouse THEMIS2 and AF647-conjugated anti-rabbit IgG were 85-340 ng and 10-50 ng, respectively.

Validation

The antibody validation for the species, the antigen specificity, and the application(s) is provided on the supplier websites and the validation statements of the manufacturers are available on the supplier websites (https://www.cellsignal.com/about-us/cst-antibody-validation-principles; https://www.thermofisher.com/jp/en/home/life-science/antibodies/invitrogen-antibody-validation.html; https://www.bdbiosciences.com/en-us/products/reagents/flow-cytometry-reagents/research-reagents/quality-and-reproducibility; https://www.biolegend.com/ja-jp/quality/quality-control; https://www.sigmaaldrich.com/JP/en/technical-documents/technical-article/protein-biology/elisa/antibody-standard-validation; https://blog.abclonal.com/blog/a-quick-guide-to-antibody-validation; and https://www.abcam.com/primary-antibodies/how-we-validate-our-antibodies). All antibodies were validated with proper isotype controls using mouse or human cells. The quality of a homemade antibody TX42.1 was validated by evaluation of the binding to primary mouse cells expressing DNAM-1 (vs. isotype control) on immune cells of wild-type and DNAM-1-deficeint mice by flow cytometry. The quality of an ATCC hybridoma-derived PK136 was validated by evaluation of the binding to primary mouse cells expressing NK1.1 by flow cytometry (vs. isotype control).

Eukaryotic cell lines

Policy information about cell lines and Sex and Gender in Research

Cell line source(s)	293F, female, Thermo Fisher Scientific, R79007<br>293gp, female, RIKEN BioResource Research Center, RCB2354<br>293T, female, ATCC, CRL-3216<br>RMA-PuroR, sex unsure, kindly provided Prof. Lewis L. Lanier (Nabekura et al. 2016)<br>RMA-m157-PuroR, sex unsure, kindly provided by Prof. Lewis L. Lanier (Nabekura et al. 2016)<br>NKL-Ly49H, male, kindly provided by Prof. Lewis. L. Lanier (Kielczewska et al. 2009)
Authentication	None of the cell lines used were authenticated.
Mycoplasma contamination	None of the cell lines were contaminated with mycoplasma.
Commonly misidentified lines (See ICLAC register)	None

Animals and other research organisms

Policy information about studies involving animals; ARRIVE guidelines recommended for reporting animal research, and Sex and Gender in Research

Laboratory animals	The following mouse strains were used in this study:<br>WT B6, CLEA Japan (Tokyo, Japan)<br>Congenic CD45.1+ B6 (B6.SJL-PtprcaPepcb/BoyJ), the Jackson Laboratory (Bar Harbor, Maine, U.S.A.)<br>Mice carrying inducible Cre expressed under the control of the Ncr1 gene harboring Rosa26-YFP alleles (NK-CreERT2 mice) on the B6 background, kindly provided by Prof. Lewis L. Lanier<br>Tyrobp-/- B6, kindly provided by Prof. Lewis L. Lanier<br>Themis2-/- B6, kindly provided by Prof. Richard J. Cornall<br>Zfp740-/- B6, generated by CRISPR/Cas9 genome editing technology in this study<br><br>Mice between 6 and 24 weeks of age were used for experiments in a gender-matched manner. All mice were housed and maintained under the specific-pathogen-free conditions (ambient temperature, 23.5oC +/- 2.5oC; humidity, 52.5% +/- 12.5%; and light cycle, 14-hour light (5 am to 7 pm)/10-hour dark (7 pm to 5 am)). The mice were fed a MF diet (MF, Oriental Yeast, Tokyo, Japan).
Wild animals	No wild animals were used in this study.
Reporting on sex	No indication that our findings apply to only one gender.
Field-collected samples	No filed-collected samples were used in this study.
Ethics oversight	All procedures were approved by the Laboratory Animal Ethics Committee of the University of Tsukuba (approval number 22-154) and performed at the laboratory animal resource centers of the University of Tsukuba in accordance with the guidelines of the institutional animal ethics committees.

Note that full information on the approval of the study protocol must also be provided in the manuscript.

Plants

Seed stocks	*Report on the source of all seed stocks or other plant material used. If applicable, state the seed stock centre and catalogue number. If plant specimens were collected from the field, describe the collection location, date and sampling procedures.*
Novel plant genotypes	*Describe the methods by which all novel plant genotypes were produced. This includes those generated by transgenic approaches, gene editing, chemical/radiation-based mutagenesis and hybridization. For transgenic lines, describe the transformation method, the number of independent lines analyzed and the generation upon which experiments were performed. For gene-edited lines, describe the editor used, the endogenous sequence targeted for editing, the targeting guide RNA sequence (if applicable) and how the editor was applied.*
Authentication	*Describe any authentication procedures for each seed stock used or novel genotype generated. Describe any experiments used to assess the effect of a mutation and, where applicable, how potential secondary effects (e.g. second site T-DNA insertions, mosiacism, off-target gene editing) were examined.*

ChIP-seq

Data deposition

☒ Confirm that both raw and final processed data have been deposited in a public database such as GEO.

☒ Confirm that you have deposited or provided access to graph files (e.g. BED files) for the called peaks.

Data access links
May remain private before publication.

H3K4me3 and H3K27ac ChIP-seq data are available in GEO with accession number GSE227138 in SuperSeries GSE227139

Files in database submission

H3K4me3 ChIP-seq data inGSE227138 in SuperSeries GSE227139: GSM7092284 to GSM7092295
H3K27ac ChIP-seq data inGSE227138 in SuperSeries GSE227139: GSM7092272 to GSM7092283

Genome browser session
(e.g. UCSC)

UCSC Genome Browser

Methodology

Replicates

ChIP-seq was performed one time with triplicated and independent samples.

Sequencing depth

Total reads: H3K4me3, 37,977,288 to 50,617,404; and H3K27ac, 34,796,452 to 49,489,508
Mapped reads: H3K4me3, 37,965,682 to 50,600,192; and H3K27ac, 34,770,400 to 49,468,882
Length of reads: 36
Sequencing: Paired-end

Antibodies

Anti-Histone H3 (acetyl K27) antibody - ChIP Grade, rabbit polyclonal Ab, Abcam, ab4729, GR3264490-1
Anti-Histone H3 (tri methyl K4) antibody - ChIP Grade, rabbit polyclonal Ab, Abcam, ab8580, GR3374555-1

Peak calling parameters

MACS3 callpeak parameters with macs3 callpeak -t treatment.bam, -c control.bam, -f BAM, --broad, -g mm, --broad-cutoff 0.1, and --SPMR

Data quality

Peaks with the cut-off FDR = 0.05: H3K4me3, 13,301 to 18,345; and H3K27ac, 14,756 to 20,764
Peaks with greater than 5-fold enrichment: H3K4me3, 8,614 to 10,385; and H3K27ac, 3,048 to 8,406

Software

Unique peaks versus input controls were identified using MACS3 (https://github.com/macs3-project/MACS). H3K4me3 DARs were extracted by bedtools on Galaxy (https://usegalaxy.org/) and these unique H3K4me3 DAR-associated single nearest genes were identified by using GREAT (http://great.stanford.edu/public/html/). H3K27ac DARs were extracted by Galaxy and then H3K27ac DARs with enhancer-like signatures (ELS) were identified by using CTCF-bound enhancer signatures available in ENCODE (https://www.encodeproject.org/) by Galaxy. These unique H3K27ac DAR-associated single nearest genes with distal and proximal ELS were identified by using GREAT. The heatmap was drawn by Morpheus (https://software.broadinstitute.org/morpheus/). These epigenome-wide heatmaps of the read density in 5 kb from TSS were drawn by deepTools on Galaxy. The heatmap of pairwise intersections was drawn with their Spearman correlations for pairwise Jaccard statistics by deepTools on Galaxy. The distance from individual memory DARs to TSS was plotted with the number of DARs within each distance range by GREAT. GO analysis of DAR genes were performed by GREAT.

Flow Cytometry

Plots

Confirm that:

☒ The axis labels state the marker and fluorochrome used (e.g. CD4-FITC).

☒ The axis scales are clearly visible. Include numbers along axes only for bottom left plot of group (a 'group' is an analysis of identical markers).

☒ All plots are contour plots with outliers or pseudocolor plots.

☒ A numerical value for number of cells or percentage (with statistics) is provided.

Methodology

Sample preparation

Blood was collected via retro-orbital venous plexus by using heparinized capillary tubes (Thermo Fischer Scientific), red blood cells were lysed with homemade ACK lysis buffer (150 mM NH4Cl, 10 mM KHCO3, and 0.1 mM Na2 EDTA at pH 7.2-7.4), washed with PBS containing 5% FCS, and passed through a mesh or strainer with 40 uM diameter. Spleens were ground by using frosted slide glasses (Matsunami Glass Industry), red blood cells were lysed with ACK lysis buffer, washed with PBS containing 5% FCS, and passed through a mesh or strainer with 40 uM diameter. In some experiments, splenocytes were incubated with biotinylated antibodies against CD4 (GK1.5), CD5 (53-7.3), CD8 (53-6.7), CD19 (6D5), Gr-1 (RB6-8C5), TER-119 (TER-119) (BioLegend) and NK cells were enriched by negative selection using Dynabeads MyOne Streptavidin C1 (Thermo Fisher Scientific). In some experiments, these cells were singly or doubly sorted with FACSAria III (BD Biosciences).

Instrument

LSRFortessa, BD Biosciences
FACSAria III, BD Biosciences

Software

BD FACSDiva V8, BD Biosciences
FlowJo V10, FlowJo

Cell population abundance

Cell populations were sorted to > 95% purity post-sort (singly sort), as determined by flow cytometry. NK cells comprised approximately 1-3% in the lymphocytes population in the blood and spleen. After NK cell enrichment, NK cells comprised approximately 40-70% in the lymphocyte population in the spleen. The purity of these cells were reported in our previous study.

Costimulatory molecule DNAM-1 is essential for optimal differentiation of memory natural killer cells during mouse cytomegalovirus infection. Immunity, 2014;40:225-34. Tsukasa Nabekura, Minoru Kanaya, Akira Shibuya, Guo Fu, Nicholas R. J. Gascoigne, Lewis L. Lanier

Gating strategy

Mouse NK cells: TCRb-NK1.1+ or TCRb-NK1.1+NKp46+ or NK1.1+TCRb-NKp46+DX5+CD49a- lymphocytes; in case NK cells in the spleen from NK-CreER2 mice after tamoxifen administration, YFP+TCRb-NK1.1+ or YFP+TCRb-B220- lymphocytes.
Human NK cells: CD3-CD19-CD56dimCD16+ lymphocytes

Viable lymphocytes were identified by FSC-SSC of the lymphocyte population at approximately FSC 50-150K-SSC 25K-150K based on FSC voltage 185-250-SSCvoltage 300-325. Doublet cells and dead cells with high autofluorescence were excluded by FSC-A-FSC-H gating, followed by SSC-A-SSC-W gating, and dead cells were excluded by using 510/50 or 525/50 nm filter of flow cytometers.

The boundary between positive and negative populations was determined based on the comparison of flow cytometric patterns stained with isotype controls or unstained controls and specific antibodies.

☒ Tick this box to confirm that a figure exemplifying the gating strategy is provided in the Supplementary Information.
